# Effects of chitosan-titanium dioxide-Glucantime nanoassemblies on *Leishmania major*: An *in vitro* and *in vivo* study

**DOI:** 10.22038/ijbms.2025.88456.19102

**Published:** 2025

**Authors:** Sanaz Tavakoli, Jaleh Varshosaz, Sedigheh Saberi, Mohammad Kazemi, Pardis Nematollahi, Nader Pestehchian

**Affiliations:** 1 Department of Parasitology and Mycology, School of Medicine, Isfahan University of Medical Sciences, Isfahan, Iran; 2 Department of Pharmaceutics, School of Pharmacy and Novel Drug Delivery Systems Research Centre, Isfahan University of Medical Sciences, Isfahan, Iran; 3 Department of Genetics and Molecular Biology, School of Medicine, Isfahan University of Medical Sciences, Isfahan, Iran; 4 Department of Pathology, School of Medicine, Isfahan University of Medical Sciences, Isfahan, Iran; 5 Infectious Diseases and Tropical Medicine Research Center, Isfahan University of Medical Sciences, Isfahan, Iran

**Keywords:** Antileishmanial therapeutics Drug delivery systems, Drug resistance in – leishmaniasis, *Leishmania major*, Leishmaniasis treatment – strategies, Titanium dioxide - nanoparticles

## Abstract

**Objective(s):**

Leishmaniasis, a neglected tropical disease, remains a public health concern. Meglumine antimonate (Glucantime®) is associated with high toxicity, prolonged treatment duration, and the emergence of drug resistance. This study aims to investigate a therapeutic strategy using chitosan-titanium dioxide-Glucantime (C-TiO₂-G) nanoassemblies comprising the natural polymer chitosan for drug loading, TiO₂ nanoparticles for enhanced cellular uptake, and Glucantime as the antileishmanial agent.

**Materials and Methods:**

Cytotoxicity was evaluated in the J774.A1 macrophage cell line to determine the IC50 values, and anti-leishmanial activity against *Leishmania major* (*L. major*) amastigotes was assessed using the Giemsa staining method. Lesion size, parasite burden, and tissue histopathology were monitored in BALB/c mice. Additionally, gene expression analysis was conducted to assess the expression of M1 and M2 macrophage polarization markers (CCR7 and CD163).

**Results:**

The nanoassemblies treatment exhibited reduced cytotoxicity with a significantly higher IC_50_ (1202.5 ± 3.5 μg/ml at 72 hr) in comparison with Glucantime (999.5 ± 3.5 μg/mL at 72 hr; *P*<0.05). Treatment with nanoassemblies (100 mg/kg) significantly reduced lesion size and parasite burden in the spleen and liver of the *L. major*-infected BALB/c mice compared with those in the negative control group (*P*<0.05). Histopathological analysis revealed less tissue damage in the liver, skin, spleen, and lymph nodes. Treatment with nanoassemblies led to immune modulation, as indicated by significant upregulation of CCR7 expression (*P*<0.0001) and downregulation of CD163 expression (*P*<0.05).

**Conclusion:**

The findings highlight the potential of chitosan-titanium dioxide-Glucantime nanoassemblies as a promising therapeutic strategy against leishmaniasis.

## Introduction

Cutaneous leishmaniasis (CL), a public health concern, is a vector-borne disease caused by protozoan parasites of the Leishmania genus, primarily found in tropical and subtropical regions (1, 2). The disease poses both diagnostic and therapeutic challenges, with clinical manifestations ranging from self-healing lesions to potentially fatal visceral infections (3). According to the World Health Organization’s 2023 surveillance report, leishmaniasis remains a significant public health issue worldwide. Globally, over the past decade, the number of reported CL cases has ranged from 185,454 in 2014 to 280,679 in 2019. Ninety-one percent of all cases were reported from Afghanistan, Algeria, Brazil, Colombia, Iran, Iraq, Pakistan, Peru, Sri Lanka, the Syrian Arab Republic, and Yemen (4). For decades, pentavalent antimonials have remained the mainstay therapy; however, their use is associated with several limitations, including high toxicity, prolonged duration of treatment, and the emergence of drug-resistant strains of the parasite. The exact mechanism through which pentavalent antimonials affect *Leishmania* remains unclear. When administered, pentavalent antimony Sb (V) undergoes bioreduction to trivalent antimony Sb (III) within *Leishmania*, initiating two main mechanisms of action. Sb (III) inhibits the enzyme trypanothione reductase (TryR), disrupting the redox balance of the parasite and elevating oxidative stress. At the same time, Sb (III) interferes with DNA topoisomerase I activity, which is crucial for the supercoiling of DNA necessary for replication and transcription. Additionally, Sb(V) exhibits its antileishmanial activity that plays a role in neutralizing reactive oxygen species (ROS), and the inhibition of TryR increases the production of ROS, resulting in additional harm to the parasite’s macromolecules, such as DNA and proteins, disrupting homeostasis and leading to the death of the parasite. The development of resistance to antimonial therapies poses a significant threat to their effectiveness, resulting in suboptimal treatment outcomes and persistent infections (5). Walker and colleagues discovered that S-adenosylmethionine synthetase (SAMS) and S-adenosylhomocysteine hydrolase (SAHH) were found to be overexpressed in Sb (III)-resistant strains and isolates, indicating a crucial role of these molecules in the resistance of *Leishmania* to Sb (6). All the abovementioned factors affect disease treatment (7-9). These limitations highlight the urgent need for novel therapeutic strategies that are more effective, safer, and capable of overcoming drug resistance (10).

Nanotechnology has emerged as a promising strategy to enhance the efficacy of conventional anti-leishmanial therapies (11). Among various nanomaterials, metal nanoparticles (NPs) exhibit broad-spectrum antimicrobial activity by interfering with microbial DNA and enzymatic functions, as well as releasing metal ions. Furthermore, they generate reactive oxygen species (ROS), damaging cell membranes (12). 

Titanium dioxide (TiO_2_) nanoparticles are particularly notable within this group. TiO_2_ nanoparticles are considered biologically safe and inert, and have been approved by the U.S. Food and Drug Administration (FDA) for application in food and pharmaceutical products (13). These nanoparticles have gained substantial attention due to their unique physicochemical properties, including a high surface area to volume ratio, biocompatibility, and photocatalytic activity. These characteristics make TiO_2_ nanoparticles especially suitable for biomedical applications, drug delivery systems, and photodynamic therapy (14).

Chitosan is a positively charged, nontoxic, biodegradable, and biocompatible biopolymer. Studies have highlighted the antimicrobial properties of chitosan; however, the precise mechanism by which it operates remains incompletely understood (15), despite three primary mechanisms being established. The first mechanism describes the interaction of NH3^+^ groups in chitosan with the negatively charged microbial cell membranes. The cell membrane becomes permeable due to this interaction, leading to microbial death. The second mechanism proposes that chitosan binds to microbial DNA, thereby blocking DNA transcription. The third mechanism demonstrates how chitosan functions as a metal chelator and essential nutrient binder, thereby preventing microbial growth. Collectively, this makes it suitable for additional research into cutaneous leishmaniasis (CL) treatment (16).

BALB/c mice are widely recognized as a well-established animal model for studying the CL, owing to their high susceptibility to *L. major* infection (17). Histopathological changes observed in infected BALB/c mice are closely associated with inflammatory responses and tissue alterations. Infected skin exhibits widespread infiltration of histiocytes, lymphocytes, and plasma cells with small granulomas formed by macrophages and lymphocytes. The lymph nodes and spleen exhibit follicular hyperplasia, sinus histiocytosis, and infiltration of lymphocytes and plasma cells. In the liver, infiltration of lymphocytes, macrophages, and plasma cells leads to sinusoidal dilatation (18). 

Histopathological manifestations in BALB/c mice with CL may vary depending on the *Leishmania* species and mouse strain. Various therapeutic strategies, including the use of plant extracts, nanoformulations, and conventional anti-leishmanial agents, have been explored for their effects on histopathological alterations in infected mice. These studies provide valuable information about the pathogenesis of leishmaniasis and the potential therapeutic efficacy of emerging treatments (3, 19, 20).

Macrophages play a dual role during *Leishmania* infection; they can either eliminate the parasite or provide a permissive environment for its growth and replication. Macrophage polarization is a critical determinant of the host immune response to *Leishmania* infection. The dynamic balance between pro-inflammatory (M1) and anti-inflammatory (M2) macrophage phenotypes significantly influences disease progression, parasite persistence, and tissue pathology (21, 22).

Based on their functional properties, macrophages are broadly categorized into two phenotypes: classically activated M1 macrophages, which exhibit cytotoxic and antimicrobial activities, and alternatively activated M2 macrophages, which display anti-inflammatory functions (23). M1 macrophages are characterized by the expression of CCR7 and the production of pro-inflammatory cytokines, including IL-23, IL-12, IL-1, and TNF-α (24). CCR7 also plays a role in the migration of memory T cells and the maturation of dendritic cells.

In contrast, M2 macrophages express CD163 and secrete cytokines such as IL-10, IL-1 receptor antagonist (IL-1RA), TGF-β, and VEGF, which are involved in promoting angiogenesis and suppression of immune responses (25). The phenotypic shift from the M1 phenotype to the M2 phenotype during *Leishmania* infection is a critical factor that influences disease progression and affects the clinical outcomes (26)**.**

Recent studies have explored nanoparticle-based therapies for leishmaniasis; however, the present study investigates explicitly nanoassemblies of TiO_2_ and Glucantime in a BALB/c mouse model. In addition, this research aims to evaluate gene expression profiles and histopathological changes, building upon findings from a previous study (27).

Nanotechnology-based approaches, particularly those utilizing metal nanoparticles, have great potential for enhancing the efficacy and safety of anti-leishmanial treatments. This integrated evaluation provides a comprehensive understanding of the therapeutic potential of such nanoformulations. To our knowledge, this research presents the first evaluation of chitosan-coated TiO₂ nanoparticles containing Glucantime (C-TiO₂-G nanoassemblies) for cutaneous leishmaniasis treatment, both *in vitro* and *in vivo*. The targeted nanoassemblies enable controlled drug delivery, enhance macrophage uptake, and strengthen the synergistic effect between the drug and photocatalyst*.* Moreover, the research investigates the immunomodulatory effects of this nanoassembly through the analysis of macrophage polarization markers, specifically CCR7 for M1 and CD163 for M2, to understand how immune modulation can enhance therapeutic results. Therefore, the current research uniquely addresses the anti-leishmanial effects of chitosan-titanium dioxide-Glucantime nanoassemblies through both *in vitro* and *in vivo *experiments. 

## Materials and Methods

### Preparation and characterization of nanoassemblies of TiO2 NPs and Glucantime

The nanoassemblies were synthesized according to our previous study (27). Briefly, nanoassemblies were prepared via electrostatic interactions and optimized using a response surface central composite design. The effects of Glucantime and TiO_2_ NPs on the particle size, zeta potential, loading efficiency, and release efficiency of the drug were investigated (27).

### Leishmania culture

The promastigotes of *L. major* (MRHO/IR/75/ER strain) were obtained from the Department of Parasitology and Mycology, School of Medicine, Isfahan University of Medical Sciences, Isfahan, Iran. Initially, parasites were cultured in Novy-MacNeal-Nicolle (NNN) biphasic medium. Upon reaching the logarithmic growth phase, they were transferred to RPMI-1640 medium (GlutaMAX Supplement, Gibco™, US) adjusted to a pH of 7.2. The medium was supplemented with 10% fetal bovine serum (FBS) (Tet system, Gibco™, UK), gentamicin (80 μg/ml), penicillin (100 IU/ml), and streptomycin (100 μg/ml). The cultures were maintained at 25 ± 1 °C for four days, and parasite growth was monitored daily.

### J774A.1 cell line culture

The J774.A1 murine macrophage cell line, obtained from the Department of Parasitology and Mycology, School of Medicine, Isfahan University of Medical Sciences, Isfahan, Iran, was cultured in RPMI-1640 medium, supplemented with 10% FBS, penicillin (100 IU/ml), and streptomycin (100 μg/ml). The cells were maintained at 37 ± 1 °C in a humidified atmosphere containing 5% CO_2_. Subculturing was performed when the cells reached approximately 70% confluency.

### Cytotoxicity assay

To evaluate the cytotoxicity of the nanoassemblies, TiO_2_, and Glucantime, J774.A1 macrophages were seeded in 96-well microtiter plates at a density of 2 × 10^4 ^cells/ml in complete RPMI-1640 medium. The cells were allowed to adhere for at least 6 hr at 37 °C in 5% CO_2_. After non-adherent cells were removed, adherent macrophages were treated with various concentrations of nanoassemblies (6.25-4000 μg/ml), TiO_2_ (6.25-4000 μg/ml), and Glucantime (100 μg/ml) in a final volume of 200 μl per well. The incubation was carried out for 24, 48, and 72 hr under the same conditions. A negative control (untreated cells) and blank wells (without cells) were included. 

Subsequently, 20 μl of sterile-filtered MTT solution (5 mg/ml, Sigma-Aldrich^®^) was added to each well, followed by incubation in the dark at 25 ± 1 °C for four hours. Subsequently, the supernatant was carefully removed, and the resulting formazan crystals were dissolved in 100 μl of dimethyl sulfoxide (DMSO). The plates were shaken for 15 min to ensure complete dissolution, and absorbance was measured at 570 nm using a microplate reader (EZ Read 400^®^, Biochrom). Cytotoxicity was evaluated by the MTT assay, and IC_50_ values were calculated using GraphPad Prism 9.0.0 (GraphPad Software, San Diego, CA, USA). All experiments were carried out in triplicate to ensure statistical robustness. The data were presented as means ± standard deviations (SDs).

### Anti-leishmanial activity against intramacrophage amastigotes assessed by giemsa staining

To evaluate the anti-leishmanial activity against intracellular amastigotes, J774.A1 murine macrophages were seeded at a density of 2 × 10^4^ cells per well in six-chamber slides (SPL, Korea) containing RPMI-1640 medium, and incubated for 6 hr at 37 °C in a 5% CO_2_ atmosphere. The macrophages were then infected with *L. major* metacyclic promastigotes at a parasite-to-macrophage ratio of 10:1, as determined using a Neubauer hemocytometer.

After 24 hr of incubation, non-internalized parasites were removed by washing with RPMI-1640 medium. The infected macrophages were subsequently treated with different concentrations (6.25 to 200 μg/ml) of nanoassemblies, TiO_2_, and Glucantime, and incubated for 24, 48, and 72 hr under the same conditions. Untreated infected macrophages served as the negative control group. 

Following incubation, the cells were fixed with absolute methanol and stained with Giemsa. The number of intracellular amastigotes per infected macrophage was counted under a light microscope and compared to that of the negative control group. Anti-leishmanial efficacy was determined by calculating the IC_50_ value, which was defined as the concentration that resulted in a 50% reduction in the number of intracellular amastigotes. All experiments were performed in triplicate to ensure statistical accuracy and reproducibility.

### In vivo experiments

Female BALB/c mice (4–6 weeks old, Australian strain) were obtained from the Razi Vaccine and Serum Research Institute, Tehran, Iran. Throughout the experiment, the mice had ad libitum access to food and water. Each mouse was subcutaneously injected at the base of the tail with 1×10^5^ metacyclic promastigotes of* L. major*. After 30 days, when visible nodules and lesions had developed, the mice were randomly assigned to nine groups (n = 6 per group).

Lesion size was measured weekly by calculating the average horizontal and vertical diameters using a Kulis Vernier caliper. The treatments were administered once daily for 28 consecutive days as follows: three groups received nanoassemblies at doses of 100, 50 and 25 mg/kg; three groups received TiO_2 _at the same respective doses; one positive control group was treated with Glucantime (100 mg/kg); a second positive control group was treated with Amphotericin B (4 mg/ml) for 21 days; and the negative control group received phosphate-buffered saline (PBS, 100 mg/ml). 

One week after the final treatment, two mice from each group were randomly chosen for lesion measurement and tissue collection. These mice were euthanized with CO_2_, and tissue samples were collected for further investigation. The remaining mice were monitored daily to assess survival rates.

### Determination of parasite burden

At the end of the experiment, two mice from each group were euthanized using CO_2_, and their livers and spleens were harvested. Impression smears were prepared and stained with Giemsa to evaluate the parasite burden. The number of amastigotes in each organ was quantified by counting parasites per 1,000 host cell nuclei and multiplying this value by the total weight of the organ (in milligrams), thus estimating the overall parasite burden. The parasite burden was subsequently assessed under an optical microscope (Olympus, Japan). 

### Histopathological evaluation

The skin, liver, spleen, and lymph node tissues of the two mice mentioned in the last section were collected and fixed in 10% neutral-buffered formalin for 24 hr. The tissues were processed for paraffin embedding using standard histological techniques. Sections (5 µm thick) were obtained using a rotary microtome and mounted on glass slides. Routine hematoxylin and eosin (H&E) staining was performed, including dehydration, clearing, and paraffin infiltration.

The stained sections were examined under a light microscope (Eclipse E800-Nikon, Tokyo, Japan) at various magnifications to assess tissue architecture, inflammatory infiltration, necrosis, and parasite presence. 

### Gene expression analysis of M1 and M2 markers

A commercial blood RNA extraction kit (Parstous, Iran) was used to extract total RNA from tissue samples, following the manufacturer’s instructions. One microgram of total RNA was reverse-transcribed into complementary DNA (cDNA) using the AddScript cDNA Synthesis Kit (Add bio, Korea) with random hexamer primers. The thermal cycling protocol for cDNA synthesis consisted of the following steps: incubation at 25 ºC for 10 min, followed by incubation at 50 ºC for 60 min, then at 80 ºC for 5 min, and finally holding at 4 ºC for 5 min. The synthesized cDNA was stored at −20 °C until further analysis. 

Quantitative real-time PCR (qRT-PCR) was performed using SYBR Green chemistry, with GAPDH used as the housekeeping gene. The primer sequences were retrieved from the NCBI GenBank database and then designed using specialized primer design software (sequences provided in Table 1). Each 10 µl reaction mixture contained 1 µl of cDNA, 5 µl of RealQ Plus 2x Master Mix Green High ROX™ (AMPLIQON, Denmark), 3.75 µl of nuclease-free water, and 0.25 µl each of forward and reverse primers.

 Amplification was performed using a StepOne™ Real-Time PCR System (Applied Biosystems, USA) under the following conditions: initial denaturation at 95 °C for 15 min, followed by 45 cycles of denaturation at 95 °C for 20 sec, annealing at 60 °C for 30 sec, and extension at 72 °C for 30 sec. All reactions were run in duplicate to ensure reproducibility. Relative gene expression levels were analyzed using the 2^-ΔΔCT^ method.

### Data analysis

The data were analyzed using Microsoft Excel 2016 and GraphPad Prism version 9.0 (GraphPad Software, San Diego, CA, USA). Two-way analysis of variance (ANOVA) was conducted, followed by Tukey’s *post hoc* test for multiple comparisons. All gene expression results are presented as the mean ± standard error of the mean (SEM). A *P*-value less than 0.05 was considered statistically significant.

## Results

### Determination of particle size and zeta potential of TiO2 nanoparticles –chitosan-Glucantime

The average size of the structures was 180 ± 19 nm, with a polydispersity index (PDI) of 0.3 ± 0.04, indicating a low diversity of particle sizes. The zeta potential of nanoassemblies of Glucantime and TiO_2_-chitosan was +45 ± 7.3 mV**.**

### Evaluation of antimony loaded in nanoassemblies of TiO2 NPs and chitosan-Glucantime

The analysis was conducted using the Optima 7300 DV ICP-OES (Inductively Coupled Plasma-Optical Emission Spectrometry) from Perkin-Elmer SCIEX, USA, at 217.582 nm. The formula G12.5C25T6 (Glucantime or G = 12.5 mg, Chitosan or C = 25 mg, TiO_2_ NPs or T = 6 mg) had 52.3% ± 1.3 of antimony concentration. 

### In vitro release of Glucantime from nanoassemblies of TiO2 NPs and chitosan-Glucantime

**Figure 1:**
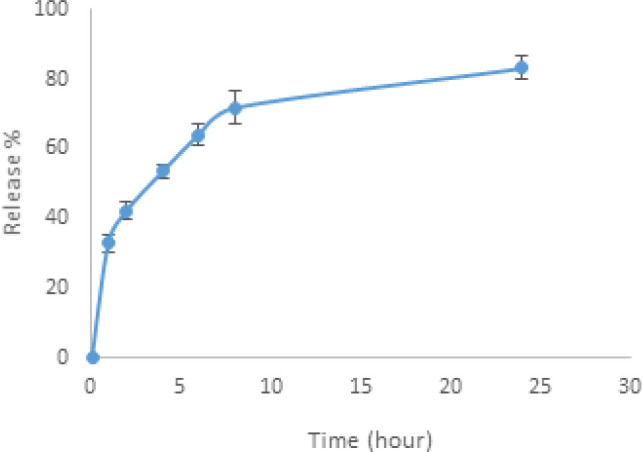
Release profile of the Glucantime from nanoassemblies of TiO2 NPs and chitosan-Glucantime

The amount of antimony released from the nanoassemblies made of TiO_2_ NPs and chitosan-glucantime was measured by ICP-OES analysis at 217.582 nm. The formulation (G12.5C25T6) released 70.35% of the antimony that was loaded in 24 hr (Figure 1). 

### Cytotoxic effects of nanoassemblies, TiO2 nanoparticles, and Glucantime on J774.A1 macrophage cell line

Treatment with the nanoassemblies resulted in a decrease in the IC_50_ values over time, from 1883.1 ± 140.0 μg/ml at 24 hr to 1606.5 ± 130.0 μg/ml at 48 hr, and then to 1202.5 ± 100.0 μg/ml at 72 hr, (*P*<0.01, 24 hr vs 72 hr), indicating increased cytotoxicity with prolonged exposure (Table 2).

Similarly, the IC₅₀ values decreased from 1608.2 ± 120.0 μg/ml (24 hr) to 1415.1 ± 110.0 μg/ml (48 hr) and 999.5 ± 85.0 μg/ml (72 hr). Compared with the Glucantime treatment, the nanoassemblies treatment resulted in significantly higher IC₅₀ values at 24 and 48 hr (*P*<0.05), suggesting lower cytotoxicity at early exposure times. However, by 72 hr, the difference was no longer statistically significant (*P*>0.05) (Table 2).

The highest IC_50_ values were obtained by TiO_2_ nanoparticle treatment throughout the experiment, with values of 4317.4 ± 300.0 μg/ml at 24 hr, 3552.2 ± 250.0 μg/ml at 48 hr, and 3290.3 ± 230.0 μg/ml at 72 hr, indicating relatively lower cytotoxicity than the other treatments (Table 2). 

### Cytotoxic effects of titanium dioxide nanoparticles, glucantime, and nanoassemblies on intramacrophage amastigotes

The efficacy of the nanoassemblies treatment was both significant and time-dependent. The IC_50_ values decreased from 64.6 ± 5.2 μg/ml at 24 hr to 31.6 ± 3.1 μg/ml at 48 hr, reaching the lowest value of 12.6 ± 1.8 μg/ml at 72 hr (*P*<0.0001 compared with the TiO_2 _and Glucantime treatments at each time point).

During TiO_2_ nanoparticle treatment, the IC_50_ values decreased from 401.2 ± 20.0 μg/ml at 24 hr to 208.9 ± 15.5 μg/ml at 48 hr and 129.4 ± 10.2 μg/ml at 72 hr. However, these values remained significantly greater than those observed for both the Glucantime and nanoassemblies treatments (*P*<0.0001).

The Glucantime treatment similarly demonstrated a reduction in IC_50_ values over time, from 65.1 ± 4.0 μg/ml at 24 hr to 43.6 ± 3.9 μg/ml at 48 hr, and 20.1 ± 2.1 μg/ml at 72 hr. While the Glucantime treatment was significantly more effective than the TiO₂ treatment at all measured time points (*P*<0.001), it remained less effective than the nanoassemblies treatment, particularly at 72 hr (*P*<0.01).

### Effect of nanoassemblies, titanium dioxide, Glucantime, and Amphotericin B treatments on lesion size

**Figure 2:**
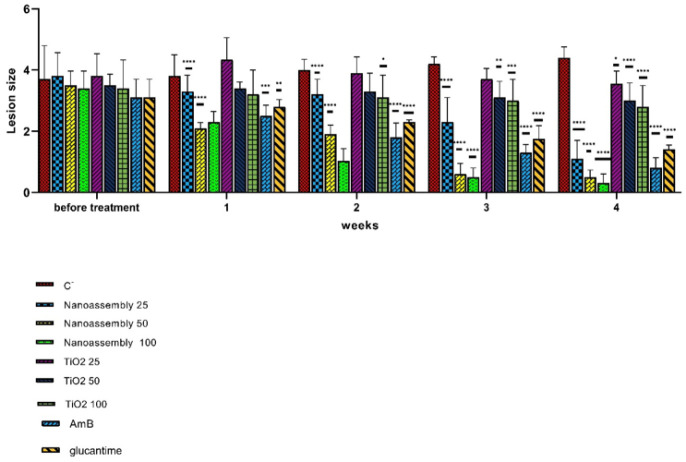
Effect of titanium dioxide nanoassembly, Glucantime, and Amphotericin B (AmB) on lesion size reduction over four weeks in intramacrophage amastigote lesions

A significant reduction in lesion size was observed within the first week in the mice treated with nanoassemblies at doses of 50 and 100 mg/kg, as well as in those treated with Glucantime and Amphotericin B, compared with the negative control group (*P*<0.05) (Figure 2). By the end of the second week, the lowest lesion size was observed in the nanoassemblies-treated group at a dose of 100 mg/kg (*P*<0.001). Additionally, significant reductions in lesion size were observed in the groups treated with nanoassemblies at 50 mg/kg, TiO_2_ at 100 mg/kg, Amphotericin B, and Glucantime (*P*<0.05). This trend continued through the third and fourth weeks, with the lowest lesion size observed in the group treated with nanoassemblies at 100 mg/kg (*P*<0.05). No significant reduction in lesion size was observed in the groups treated with TiO_2_ nanoparticles at doses of 25 and 50 mg/kg (*P*>0.05). 

### Effect of nanoassemblies, titanium dioxide, glucantime, and Amphotericin B treatments on parasite burden

**Figure 3.**
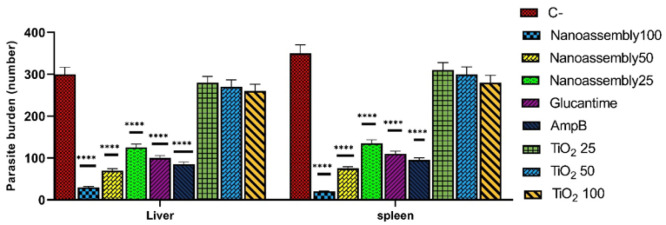
Effects of Nanoassemblies, Titanium Dioxide, Glucantime, and Amphotericin B on intramacrophage amastigotes: parasite burden in the liver and spleen

The effects of different treatments on the parasite burden in the liver and spleen are presented in Figure 3. The highest parasite burdens were reported in the negative control group for both organs. The nanoassemblies treatment at a dose of 100 mg/kg significantly reduced the parasite burden in the liver and spleen compared to the negative control group (*P*<0.0001). This reduction was significantly greater than that observed in the Glucantime group (*P*<0.001) and the Amphotericin B group (*P*<0.01). The nanoassembly treatment at a dose of 50 mg/kg also reduced the parasite burden compared to the negative control group (*P*<0.0001), although it was slightly less effective than the 100 mg/kg dose (*P*<0.05). The 25 mg/kg dose also reduced parasite burden (*P*<0.001), which was the least effective among the other doses (*P*<0.01). In contrast, TiO_2_ nanoparticles at doses of 25, 50, and 100 mg/kg did not significantly reduce the parasite burden (*P*>0.05).

### Effects of nanoassemblies, titanium dioxide, glucantime, and Amphotericin B treatments on the survival rate

**Figure 4:**
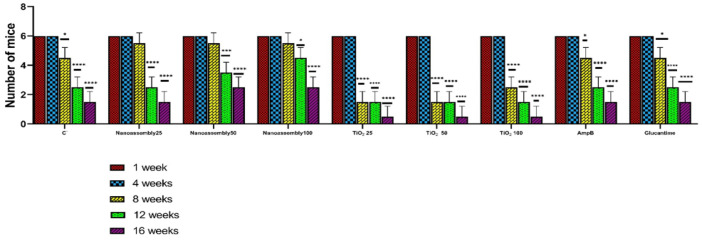
Effects of various treatments on the number of mice with intramacrophage amastigotes at different weeks

The number of surviving mice decreased over time across all treatment groups. The highest survival rate was reported in the nanoassemblies treatment group, which received 100 mg/kg nanoassemblies throughout the experimental period, compared with the negative control group. Both groups initially included six mice at week 1 (*P*>0.05). No mortality was recorded in the nanoassembly treatment group at week 4 (*P*<0.05). The survival rate remained significantly greater in this group at week 8 (*P*<0.05), week 12 (*P*<0.001), and week 16 (*P*<0.0001), with three mice surviving, whereas no mice remained in the negative control group (Figure 4). Among the groups treated with TiO_2_, the 100 mg/kg dose was associated with the most pronounced reduction in survival during the observation period. Higher survival rates were observed in the Amphotericin B and Glucantime treatment groups than in the negative control group. By week 16, two mice remained alive in each of these groups, whereas all mice in the negative control group had died (*P*<0.0001).

### Histopathological findings

*Liver*

**Figure 5:**
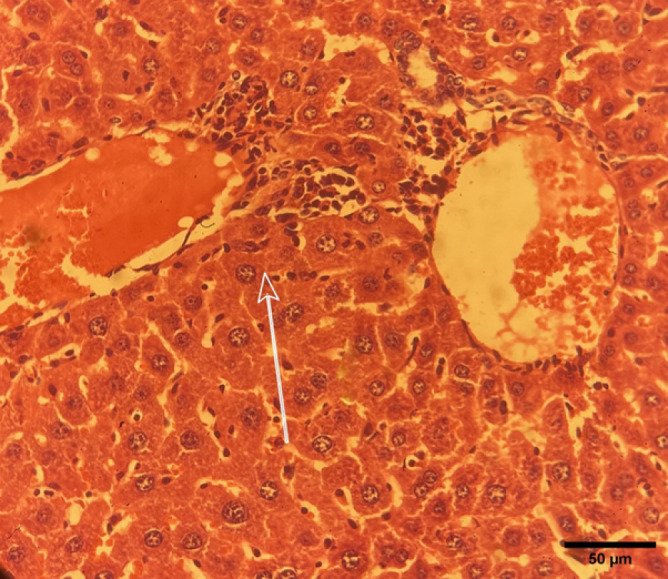
Histopathological changes in the liver of the negative control group mice

Histopathological analysis of liver tissues from the negative control group was performed under a microscope. The liver parenchyma displayed polygonal cells with centrally located nuclei, characteristic of liver hepatocytes. Lobular organization with hepatocytes surrounding the vascular structures of the liver tissue is indicated (Figure 5). A blood vessel, possibly a branch of the portal vein, filled with red blood cells was observed. There were some spaces between hepatocytes that corresponded to the sinusoids, where Kupffer cells (liver macrophages) were present. There was an accumulation of inflammatory cells around the portal areas, suggesting portal inflammation, which was indicative of infection. Additionally, focal fibrosis of hepatocytes was observed (Figure 4). The other therapeutic interventions did not result in any notable pathological changes. 

*Skin*

**Figure 6:**
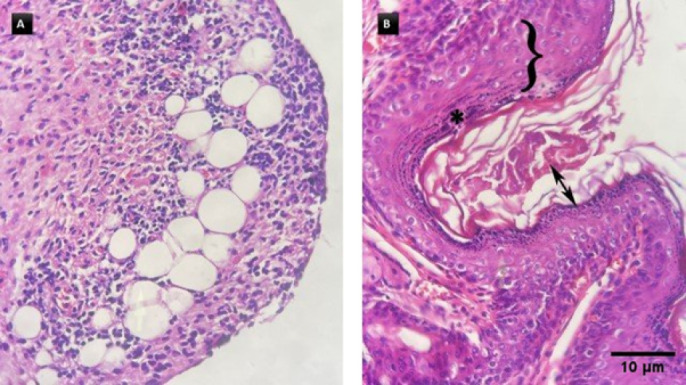
Histopathological changes in the skin tissue of negative control mice

In the negative control group, remodeling of the connective tissue was indicated in the dermis, indicating chronic inflammation and infiltration of inflammatory cells (Figure 6A). Signs of hyperkeratosis and acanthosis, together with hypergranulomatosis and ulceration of the thickened epidermis, were reported in the negative control group (Figure 6B). In the TiO2-treated group, infiltration of inflammatory cells, including lymphocytes, macrophages, and plasma cells, was observed, accompanied by a loosely organized dermal structure, which suggests tissue damage or remodeling processes. In the Glucantime-treated group (positive control), hyperplasia or reactive changes were observed in the thickened epidermis. Signs of inflammatory cell accumulation have been reported in dermal tissue. No distinct pathological changes were observed in the nanoassembly treatment group.

*Spleen*

**Figure 7:**
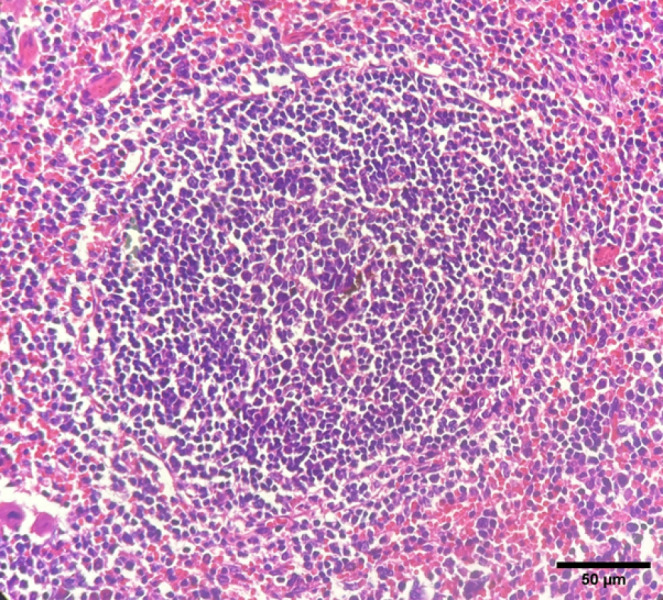
Histopathological changes in the spleens of negative control mice

Perifollicular fibrosis was detected in the negative control group (Figure 7), whereas no signs of follicular fibrosis were reported in the positive control group. Evidence of reactive hyperplasia or fibrosis was observed in the spleens of the mice treated with TiO_2_. In the group treated with nanoassemblies, lymphoid follicles were normal in size, with no signs of follicular hyperplasia or fibrosis.

*Lymph node*

**Figure 8:**
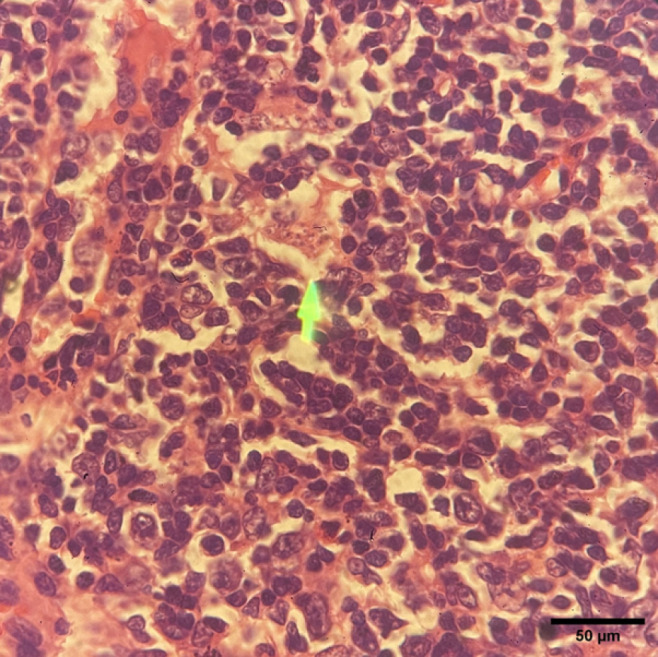
Histopathological changes in the lymph nodes of the negative control group mice

In the negative control group, the lymph node architecture appeared preserved, with numerous small, dark-stained lymphocytes indicating a typical lymphoid structure. No significant architectural features were observed, and there was no evidence of granuloma formation, fibrosis, or necrosis. Macrophages containing amastigotes were detected (Figure 8).

### Expression of the CCR7 gene in lesions

**Figure 9:**
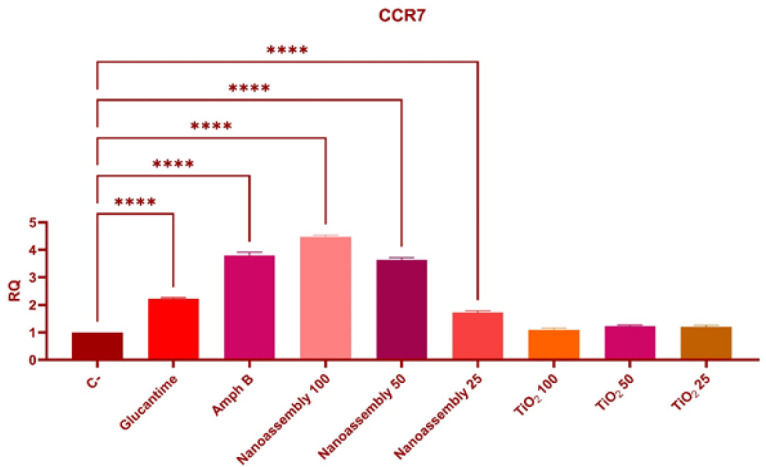
Expression of the CCR7 Gene in lesions: Differential impact of various treatments

Treatment with Glucantime significantly up-regulated the expression of the CCR7 gene compared to the negative control group (2.21 ± 0.05 vs 1.00 ± 0.00, *P*<0.0001) (Figure 9). Similar up-regulation was observed in the group treated with Amphotericin B (3.80 ± 0.12, *P*<0.0001). Among all the experimental groups, the nanoassembly administered at a dose of 100 mg/kg induced the highest level of CCR7 expression (4.47 ± 0.06 vs the control, *P*<0.0001). Compared with the control group treatment, the 100, 50, and 25 mg/kg TiO_2_ treatments did not result in statistically significant changes (1.09 ± 0.06, 1.23 ± 0.03, and 1.20 ± 0.06, respectively;* P*>0.05). 

### Expression of the CD163 gene in lesions

**Figure 10:**
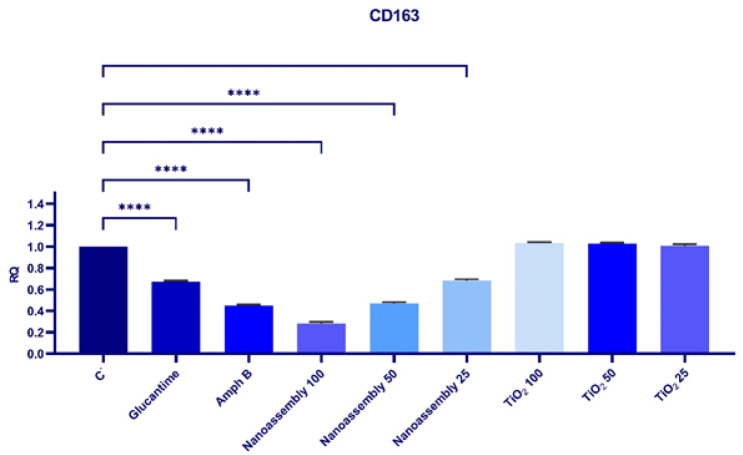
Expression of the CD163 gene in lesions: Differential impact of various treatments

Treatment with Glucantime resulted in a significant down-regulation of CD163 gene expression compared to the control group (0.67 ± 0.01 vs 1.00 ± 0.00*, P*<0.05). A more pronounced decrease was observed in the Amphotericin B-treated group (0.45 ± 0.01, *P*<0.05). Nanoassembly at 100 mg/kg caused a significant reduction in CD163 expression (0.28 ± 0.01, *P*<0.05*)*. Low doses of the nanoassemblies (50 mg/kg and 25 mg/kg) also significantly decreased the expression levels to 0.47 ± 0.01 and 0.68 ± 0.01, respectively (*P*<0.05). In contrast, TiO_2_ treatments at 100 mg/kg and 50 mg/kg did not result in any significant change in the expression of CD163 compared with that in the control group (1.03 ± 0.01 and 1.02 ± 0.01, respectively; *P*>0.05) (Figure 10).

## Discussion

The present study aimed to assess the cytotoxic and therapeutic effects of titanium dioxide (TiO_2_) nanoparticles, Glucantime, and nanoparticle-drug assemblies in the treatment of *Leishmania* infection. An *in vitro* cytotoxicity assay was conducted using the J774.A1 macrophage cell line, and the effectiveness of anti-leishmanial treatments was evaluated across different concentrations and time intervals. Furthermore, gene expression analysis related to M1 and M2 macrophage polarization was conducted.

The growing antimonial resistance in endemic areas of the Indian subcontinent and South America creates an urgent need for new therapies that are both less toxic and support the immune system (28). The present research provides insights into the medical applicability and safety profiles of the evaluated treatments. Among the tested treatments, the nanoassemblies exhibited the lowest cytotoxicity, with the highest IC_50_ values at each time point. TiO_2_ nanoparticles demonstrated the lowest cytotoxicity and the highest IC_50_ values, signifying a favorable safety profile compared to other treatments. In contrast, Glucantime displayed greater cytotoxicity than the other nanoassemblies. The increase in cytotoxicity over time is the key factor in determining the optimal dosage and treatment duration.

The efficacy of nanoassemblies treatments was reported as the IC_50 _values decreased over the treatment period. Glucantime treatment and nanoassemblies treatments were comparable in terms of efficacy, with a slight difference; nanoassemblies treatments yielded better results. The current research, consistent with earlier studies, emphasizes the cytotoxicity of Glucantime, underscoring the urgent need for safer therapeutic alternatives (29). Although TiO_2 _nanoparticles exhibited anti-leishmanial activity, their efficacy was lower than that of the nanoassemblies and Glucantime. TiO_2 _nanoparticles are reported to have a promising safety profile, supporting their potential effect in prolonged treatments​ (30). 

Recent findings indicated that nanoassemblies induced a time-dependent reduction in IC_50_ values, effectively inhibiting the growth of *Leishmania* parasites. These results are further supported by advancements in nanoparticle-based therapies, such as the use of Ag NPs, which display anti-leishmanial properties attributed to their unique characteristics and high surface area. The Ag NPs exhibited significant activity against *Leishmania* after 24 hr of exposure, with IC_50_ values of 14.94 and 3.89 μg/ml in promastigotes and intracellular amastigotes, respectively. (31). Moreover, the efficacy of curcumin-loaded mannose-functionalized chitosan nanoparticles (Cur-MCNs) was comparable to or even superior to that of Glucantime. An *in vitro* cytotoxicity study of the J774A.1 macrophage cell line revealed its non-toxicity toward macrophages. *In vivo* anti-leishmanial activity in hamsters resulted in significantly greater suppression of parasite replication in the spleen with Cur-MCNs than with chitosan nanoparticles (32).

The therapeutic effects on lesion size were assessed over a four-week period. Among the tested treatments, the nanoassemblies, Glucantime, and Amphotericin B led to reductions in lesion size; however, the nanoassemblies had the most significant effect. These treatments also affected the parasite burden in both the spleen and liver. Compared with the negative control group, both nanoassemblies and Glucantime treatments resulted in a significant reduction in the parasite burden within these organs. Amphotericin B also exhibited anti-leishmanial efficacy, although to a lesser extent than the nanoassemblies.

A study revealed that the mean number of parasites and the mean diameter of the lesions were considerably decreased (*P*<0.05) in infected mice using synthesized PO-coated iron oxide magnetic nanoparticles (Fe3O4@PO NPs), suggesting a promising outlook for treatment strategies (33). Furthermore, significant reductions in the number of parasites were observed when nanoparticle-Glucantime combinations were used; this result aligns with the findings of previous studies (34, 35). 

In another study, a transdermal formulation in the form of a nanostructured lipid carrier (NLC)-based hydrogel containing Glucantime exhibited significantly enhanced anti-leishmanial efficacy. Treatment with this nanoformulation resulted in a significant reduction in wound size and a decrease in parasite number in lesions, the liver, and the spleen when compared to Glucantime administration (35).

The present research evaluated the histopathological alterations in the liver, skin, spleen, and lymph nodes of CL-infected mice, comparing untreated controls with TiO₂-treated, Glucantime-treated, and nanoassembly-treated groups. This research was carried out to reveal tissue-specific responses to both infection and treatment; it also aimed to evaluate the potential of nanoassemblies in mitigating disease-related pathology. 

The liver tissue showed a normal lobular structure, with polygonal hepatocytes maintaining their typical cord arrangement. The portal triad structure is clearly defined through its bile duct, portal vein, and hepatic artery elements, which illustrate the structural integrity of the liver. The portal area showed inflammatory cell infiltration that may have represented an early or controlled pathological response. The observed histological changes were consistent with those observed in hepatic infections and inflammatory conditions, as initial immune responses typically present as mild inflammation before progressing into fibrosis or necrosis (36). 

One study reported that *Leishmania* infection resulted in liver inflammation and necrosis due to systemic parasite dissemination (20). In the current study, Kupffer cells were located in sinusoidal spaces, suggesting that active immune surveillance serves as a fundamental defense mechanism of the liver against pathogens. The immune system employs Kupffer cells to perform phagocytosis and antigen presentation, which helps control immune responses during leishmaniasis (37).

The histological analysis of skin tissue from the negative control group revealed marked evidence of inflammation, indicating an active and ongoing immune response. The presence of spindle-shaped cells, together with extracellular matrix deposition, points to fibrotic remodeling —a possible outcome of chronic inflammation associated with granuloma formation in cutaneous infections (38). The co-occurrence of hyperkeratosis and dermal remodeling further supported the presence of chronic inflammation driven by persistent *Leishmania* infection. The histological features confirmed those of a previous study on the CL, in which dermal macrophage infiltration and amastigote identification served as essential diagnostic markers (39). 

In the TiO_2_-treated group, increased accumulation of inflammatory cells, along with granuloma development, was observed. Glucantime-treated mice exhibited moderate epidermal thickening and reduced dermal inflammation, indicating partial therapeutic efficacy. The nanoassembly treated group presented minimal epidermal changes and preserved normal dermal structure, indicating a successful reduction in inflammatory responses.

Histological examination of the spleens of the negative control mice revealed typical features of immune activation, including varying degrees of lymphoid follicle development. The presence of follicular hyperplasia indicates that the immune system’s activity increases, which typically occurs during infection (40). On the contrary, splenic sections without significant hyperplasia indicated variability in immune responses, potentially affected by host-specific factors including genetic predisposition, infection duration, or pathogen-induced immune modulation. The observation of peri-follicular fibrosis further supported the presence of chronic immune activation. This condition typically arises from persistent inflammation, which leads to excessive collagen deposition due to continuous immune stimulation (41). The observed fibrosis, in line with previous findings, confirmed that prolonged immune responses led to splenic fibrotic changes (42). 

In the TiO_2_-treated group, the enlargement of the white pulp and fibrotic changes indicated ongoing and possibly exacerbated immune activation. Nanoassembly treatment preserved the normal splenic architecture, whereas Glucantime-treated mice exhibited low degrees of fibrosis. Spleens from nanoassembly-treated mice presented no signs of fibrosis or hyperplasia, demonstrating the potential protective effects of nanoassemblies on splenic tissue.

The lymph nodes of the negative control group exhibited a typical architecture, characterized by an abundance of lymphocytes and the absence of granulomas or fibrosis. The detection of intracellular amastigotes inside macrophages confirmed active *Leishmania* infection, which is consistent with the known pathogenesis of CL (43). 

Comparative histopathological assessments across treatment groups underscored the efficacy of nanoassemblies in mitigating CL-induced tissue damage. Treatments with TiO_2_ and Glucantime resulted in varying degrees of inflammation and fibrosis; however, the nanoassemblies effectively preserved tissue architecture in the liver, skin, spleen, and lymph nodes. These findings suggest that nanoassemblies not only target the parasite but also modulate the host immune system’s response to prevent excessive tissue remodeling.

The expression levels of the CCR7 and CD163 genes in Leishmania-induced lesions were evaluated to assess the immunomodulatory effects of the treatments. Notably, the expression of CCR7 was significantly up-regulated following nanoassemblies treatments, suggesting a potential immunostimulatory effect. This upregulation could strengthen the immune system’s response against infection when nanoassemblies were used at a concentration of 100 µg/ml. Amphotericin B treatment also triggered an up-regulation in CCR7 expression. The observed increase in CCR7 indicates that dendritic cell maturation and their migration to lymphoid organs have improved, which could result in better T-cell activation. The nanoassembly group shows improved lesion clearance and reduced systemic parasite burden, which could be explained by this mechanism. The decrease in CD163, a marker associated with tissue repair and immunosuppression, suggests that nanoassemblies may prevent chronic infections by blocking M2-related pathways (21).

Floro *et al*. (44) investigated macrophage polarization during *Leishmania* (*Viannia*) *braziliensis* infection in mice. They reported that during infection, the proportion of M2 macrophages in different tissues was significantly greater than that of M1 macrophages. These findings confirmed that *Leishmania* promotes and affects macrophage phenotype polarization in various tissues. Similarly, Emerson *et al*. (45) indicated that extracellular vesicles (EVs) released by *Leishmania* induce the transcriptional activation of genes associated with M2 macrophages. These findings also highlighted the role of M2 macrophages in anti-inflammatory responses and tissue remodeling. 

The expression of the CD163 gene, which is commonly associated with anti-inflammatory responses and M2 macrophage polarization, was significantly reduced following treatment with nanoassemblies. The most significant down-regulation was recorded in the nanoassemblies-treated group, while the TiO_2_ treatment had no significant effect. 

These findings highlight the potential of these therapeutic agents to influence macrophage polarization and modulate the immune system’s response. These results are in accordance with previous research, which indicates that nanoparticle-based therapies can modulate immune function by altering gene expression profiles and enhancing immune cell activation, thereby improving anti-leishmanial efficacy (46).

Despite providing valuable insights, this study is limited by its reliance on *in vitro* and animal models, which may not entirely replicate the intricate interactions present in human infections. Future research should prioritize clinical trials to validate these findings and evaluate the long-term safety and effectiveness of these treatments. Additionally, Future research needs to include pharmacokinetic profiling, long-term safety assessments, and *in vivo* efficacy in immunocompromised models to establish clinical relevance. The potential therapeutic benefits of nanoassemblies could be increased by studying their synergistic effects with other immunostimulatory agents.

**Table 1:** The sequences of primers used for real-time PCR to analyze gene expression in BALB/c mice (Mus musculus)

**Gene name**	**Primer sequences**
**CCR7**	Forward primer CAGGTGTGCTTCTGCCAAGAT
	Reverse primer GGTAGGTATCCGTCATGGTCT
**CD163**	Forward primer GGTGGACACAGAATGGTTCTTC
	Reverse primer CCAGGAGCGTTAGTGACAGC
**GAPDH**	Forward primer AGGTCGGTGTGAACGGATTTG
	Reverse primer TGTAGACCATGTAGTTGAGGTCA

**Table 2:** IC50 (μg/ml) ± SD values of various treatments for J774.A1 cell line macrophages (24 hr, 48 hr, 72 hr)

	IC_50_ (μg/ml) ±SD		
	24 hr	48 hr	72 hr
Nanoassemblies	1883.1 ± 140.0	1606.5 ± 130.0	1202.5 ± 100.0
TiO_2_	4317.4 ± 300.0	3552.2 ± 250.0	3290.3 ± 230.0
Glucantime	1608.2 ± 120.0	1415.1 ± 110.0	999.5 ± 85.0

**** *P*<0.0001, *** *P*<0.001, ** *P*<0.01, * *P*<0.05

**Table 3:** IC_50_ (μg/ml) ± SD values of various treatments for intramacrophage amastigotes (24 hr, 48 hr, 72 hr)

	IC_50_ (μg/ml) ±SD		
	24 hr	48 hr	72 hr
Nanoassemblies	64.6 ± 5.2	31.6 ± 3.1	12.6 ± 1.8
TiO_2_	401.2 ± 20.0	208.9 ± 15.5	129.4 ± 10.2
Glucantime	65.1 ± 4.0	43.6 ± 3.9	20.1 ± 2.1

**** *P*<0.0001

**** P<0.0001, *** P<0.001, ** * P*<0.01, * * P*<0.05

Hematoxylin-eosin (HE) staining (magnification ×40), white arrow: accumulation of inflammatory cells around the portal area, scale bar = 50 μm

A: Infiltration of inflammatory cells. B: Double-headed arrow: hyperkeratosis, bracket: acanthosis, star: hypergranulomatosis. Hematoxylin-eosin (HE) staining (Magnification ×40), scale bar = 10 μm

Hematoxylin-eosin (HE) staining (magnification ×40), scale bar = 50 μm

Arrow: amastigote. Hematoxylin-eosin (HE) staining (magnification ×40), scale bar = 50 μm

The data are presented as the means ± SEMs, and P<0.05 was considered statistically significant. **** *P*<0.0001.

The data are presented as means ± SEMs, and *P*<0.05 was considered statistically significant. **** *P*<0.0001

## Conclusion

The present study investigated the therapeutic potential of nanoassemblies, TiO_2_, and Glucantime in treating *Leishmania* infections, with a focus on balancing cytotoxicity effects and therapeutic efficacy. Nanoassemblies demonstrated superior efficacy, significantly modulating immune responses while maintaining a favorable safety profile. TiO_2_ treatment resulted in minimal cytotoxicity, indicating that it is a safer option, albeit it is a less potent treatment. The up-regulation of CCR7 and down-regulation of CD163 explain the enhanced immune activation and reduced anti-inflammatory effects, contributing to more effective infection control. Studying gene expression profiles confirms that nanoparticle-based treatments induce effects that could underlie their improved therapeutic outcomes.

## Declaration

We have not used any AI tools or technologies to prepare this manuscript.
